# Content-Sensitive Characterization of Peer Interactions of Highly Engaged Users in an Online Community for Smoking Cessation: Mixed-Methods Approach for Modeling User Engagement in Health Promotion Interventions

**DOI:** 10.2196/jopm.9745

**Published:** 2018-07-24

**Authors:** Sahiti Myneni, Vishnupriya Sridharan, Nathan Cobb, Trevor Cohen

**Affiliations:** ^1^ School of Biomedical Informatics University of Texas Health Science Center at Houston Houston, TX United States; ^2^ Georgetown University Medical Center Washington, DC United States

**Keywords:** user engagement, smoking cessation, text analysis

## Abstract

**Background:**

Online communities provide affordable venues for behavior change. However, active user engagement holds the key to the success of these platforms. In order to enhance user engagement and in turn, health outcomes, it is essential to offer targeted interventional and informational support.

**Objective:**

In this paper, we describe a content plus frequency framework to enable the characterization of highly engaged users in online communities and study theoretical techniques employed by these users through analysis of exchanged communication.

**Methods:**

We applied the proposed methodology for analysis of peer interactions within QuitNet, an online community for smoking cessation. Firstly, we identified 144 highly engaged users based on communication frequency within QuitNet over a period of 16 years. Secondly, we used the taxonomy of behavior change techniques, text analysis methods from distributional semantics, machine learning, and sentiment analysis to assign theory-driven labels to content. Finally, we extracted content-specific insights from peer interactions (n=159,483 messages) among highly engaged QuitNet users.

**Results:**

Studying user engagement using our proposed framework led to the definition of 3 user categories—conversation initiators, conversation attractors, and frequent posters. Specific behavior change techniques employed by top tier users (threshold set at top 3) within these 3 user groups were found to be goal setting, social support, rewards and threat, and comparison of outcomes. Engagement-specific trends within sentiment manifestations were also identified.

**Conclusions:**

Use of content-inclusive analytics has offered deep insight into specific behavior change techniques employed by highly engaged users within QuitNet. Implications for personalization and active user engagement are discussed.

## Introduction

With the increasing recognition of the importance of participatory medicine in care delivery, researchers seek to improve traditional health care by placing the health consumer at the center of the system by means of “connected health” [1]. Biosensing devices, social media, big data analytics, treatment modalities, and patient engagement pathways define the infrastructure underlying connected health implementations [2,3]. The Web of intelligent communication and actionable insights can enable integrated care and better outcomes for health consumers [4]. Empowered and engaged health consumers (and patients) in conjunction with technological advances can create a more streamlined and efficient system from the current health sector, which is chaotic and fragmented [1]. To this end, social media and online communities (often referred to as the “participative internet” [5]) are gaining popularity as venues for management of health and wellness. Traditionally, health care services and clinicians act as intermediaries in providing health consumers and patients with relevant and vetted health information. However, with the penetration of consumer-driven Web, these new generation platforms have been gaining popularity as predominant information sources where social contacts, collaborative tools (eg, content recommendation systems) and agents (eg, virtual assistants) guide health consumers and patients toward adoption and maintenance of positive health behaviors [6]. Accessibility, scalability, and affordability are key characteristics that can make these platforms effective public health interventions for health promotion and behavior change [7]. In order to achieve the full potential of these platforms, we need to address challenges related to adoption and sustained use [8-10]. Understanding the facets of user engagement in an online health community can help us drive acceptance and enhance utility of online social platforms in health care. Strong user engagement with a technology intervention may enhance outcomes [8]. Health consumers use internet-based resources differently than they do in-person and group programs, and hence, the characteristics of subpopulations must be assessed to determine the factors leading to sustained use [11]. Further, such nuanced understanding of patterns of use can help us optimize care pathways and personalize design of technologies to achieve long-term engagement [12].

There are multiple definitions of user engagement in health care settings [13-17]. As defined by Lalmas [17], user engagement is “the quality of user experience that emphasizes the desire of a user to use the application longer and in frequent intervals.” Traditionally, user engagement metrics are calculated by number of page views or time spent on specific activities [18]. In the context of a social platform, however, the volume of posts (also known as communication frequency) is the most used criterion to describe levels of user engagement in both short-term and long-term use. This quality of user experience can be described using a complex set of factors that are emotional, cognitive, and behavioral in nature [13]. Existing metrics do not capture this holistic view of user engagement with a technological resource such as an online social platform.

Previous studies on user engagement in online communities have focused on (1) enhancing user engagement through gamification and related techniques [19], (2) estimating the impact of personalization on engagement [20], (3) studying engagement from the perspective of the 3 main types of social support—informational support, emotional support, and companionship [21], (4) studying user engagement from the perspective of user roles such as lurkers [22,23] and user evolution across these roles [8], (5) estimating engagement through data logs [24], and (6) studying social bootstrapping and social curation as possible aspects of improved user engagement [25,26]. On the other hand, our prior research on online social platforms has highlighted the need to consider content of user communication to better characterize peer interactions [27-30]. The content itself can have a strong influence on user engagement [27]. Also, sustained engagement is likely when the intervention offers ongoing learning and interactive opportunities. The extent to which these requirements are fulfilled can be understood by studying the content of communication from a theoretical perspective [30]. However, the dynamics of content-based attributes and user engagement in online communities has rarely been studied together.

In our study, we aimed to address these gaps by developing new methods inclusive of communication frequency and communication content, thus enabling the study of user engagement in online health communities. The relationship between these communication attributes and user engagement is detailed in the literature [5,20,31,32]. The methodology described in the paper takes an integrated approach from various user engagement models, some of which are specific to online communities for health behavior change [31,32]. We apply this proposed new model to analyze peer interactions in QuitNet, an online community for smoking cessation.

## Methods

### Materials

QuitNet is one of the first online communities for behavior change with historically over 100,000 new registrants per year [33]. Previous studies show that participation in QuitNet is associated with abstinence [34]. In this paper, we examined a data set from a version of QuitNet that ran until approximately 2015 where the primary mode of communication was threaded forums; in other words, peer conversations were initiated with an initial message and replies were displayed hierarchically. Each forum message is identified using a message ID, a thread ID, a sender ID, and a recipient ID. Participants set quit dates which represent abstinence from smoking and are preserved in historical logs if they change. This research project was reviewed and exempted by the Institutional Review Board at the University of Texas Health Science Center at Houston.

### Methods

#### Characterization of QuitNet Users

We defined 3 QuitNet user groups based on the frequency in which users engaged in peer interactions within QuitNet. Empirical cut points based on previous research were used to define the groupings as described below:

- Conversation initiators: users who initiated the highest number of threads
- Frequent posters: users who posted the highest number of messages to the forums
- Conversation attractors: users whose posts attracted the most replies

For the purpose of this study, our analysis included the top 3 QuitNet users within each user group (that is, 9 users in 3 groups in a given year) across the years 2000 to 2015, resulting in 144 unique users. Overall, 26,466 messages were exchanged by conversation attractors, 57,379 messages were exchanged by conversation initiators, and 75,638 messages were exchanged by frequent posters. In total, these 144 highly engaged users exchanged 159,483 messages. Further, we analyzed the user demographics (age, gender) and self-reported smoking status where available in event logs. For users whose event logs were incomplete or unavailable (42.4% [61/144] of the users), we analyzed their messages manually, leveraging the fact that QuitNet users specify the number of days since they last smoked in every message as a form of tradition. From these data, we estimated user abstinence status across years and identified users as falling into one of the following categories: (1) abstinent for less than 3 months, (2) abstinent between 3 and 6 months, (3) abstinent between 6 months and 1 year, (4) abstinent between 1 and 2 years, (5) abstinent for more than 2 years, and (6) active smoker.

#### Characterization of QuitNet Communication Content

We analyzed 2.05 million messages generated by 102,005 unique users, exchanged between the years 2000 and 2015 for this purpose. Using a series of qualitative and automated text analysis methods, we categorized QuitNet messages into 16 themes.

##### Qualitative Analysis

**Figure 1:**
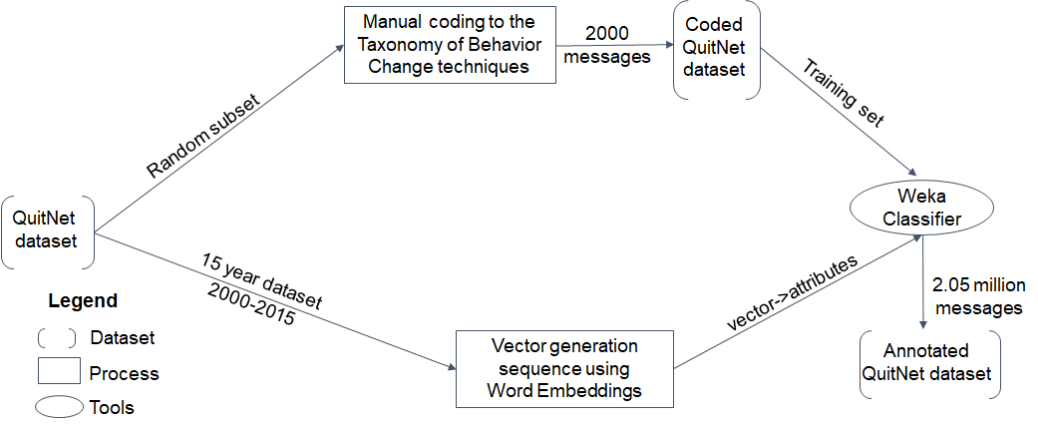
Methodological outline for content analysis.

We selected 2000 messages at random to produce an annotated subset of QuitNet messages (Figure 1). These messages were manually coded using the taxonomy of behavior change techniques [35] by 2 independent coders. The taxonomy has 93 theoretically linked behavior change techniques clustered into 16 thematic categories that were developed by a team of behavior change experts and drawn from multiple behavior change theories such as social change theory and the health belief model. The list of techniques of the taxonomy with definitions and subcategories can be found in Michie et al [35]. Table 1 shows snippets of sample messages from QuitNet that correspond to a particular technique of the taxonomy. From the sample messages, it can be seen that a single message in QuitNet can be mapped to multiple techniques of the taxonomy. The interrater reliability was estimated at 0.76 for the 2 coders during annotation. Most disagreements were due to multiple labels assigned to each message. All coding conflicts were resolved through discussion before the manual annotation of QuitNet messages was finalized.

##### Automated Text Analysis

**Figure 2:**
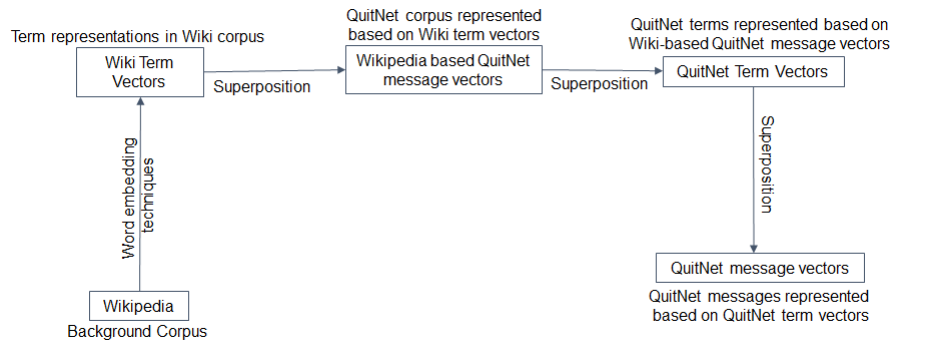
Vector generation sequence of QuitNet messages.

All messages in the dataset were annotated by providing the QuitNet vector representations to a machine learning classifier trained on the manually annotated messages. The components of the process are represented in Figure 2. To generate vector representations of messages, we used neural word embeddings, specifically the Skipgram-with-Negative-Sampling (SGNS) algorithm developed by Mikolov and colleagues [36], as implemented in the open source semantic vectors [37] package for distributional semantics. With SGNS, a neural network is trained to predict the probability of encountering terms that occur in proximity to an observed term. For example, one might anticipate a relatively high probability of observing the term “relaxing” in proximity to the term “hammock.” During the course of training, the neural network learns to predict higher probabilities for observing contextual terms that are observed in proximity to a term in the corpus than it does for those that are not observed in proximity to this term. Each term is associated with a set of weights—a weight vector—that encodes the terms that have been observed in proximity to it. Terms that occur in similar contexts will have similar weight vectors, and the similarity between the resulting weight vectors has been shown to correlate well with human estimates of the similarity between concept pairs. An advantage of this approach is that it permits the incorporation of background knowledge into a categorization model. For example, if 2 QuitNet users refer in messages to a concept such as “sadness” with different terms (such as “feeling blue,” “miserable today”), a classifier will be able to assign a category attached to one of these messages from the training set to the other previously unseen message because the words in these messages have similar representations, even though they are not identical words. Information of this sort can be learned by SGNS from a large unannotated general domain corpus.

For our research, Wikipedia was used as a background corpus. Our Wikipedia corpus contains 1.9 billion words in more than 4.4 million articles, and 500-dimensional Wikipedia-derived term vectors were obtained by applying the SGNS algorithm to the Wikipedia background corpus. This decision was motivated in part by the terse nature of the messages exchanged in QuitNet user forums, which often do not provide enough contextual information to train a distributional model [28]. However, there are ways in which the language used in QuitNet differs from that used in the Wikipedia. Of particular importance for the current research, QuitNet messages contain neologisms, community-specific terms such as “nicodemon” and “sickarettes.” While these terms are unlikely to appear in Wikipedia, within QuitNet they occur in the context of other terms for which robust representations have been learned from this larger corpus. As such, the distributional information learned from Wikipedia can be viewed as a form of prior knowledge, providing a starting point for the next phase—distributional modeling of the QuitNet corpus. This component of the training process used an iterative training procedure [38] in which term and message vectors are generated in succession.

Specifically, we first superposed (added together) the Wikipedia term vectors for the terms that occur in each QuitNet message to obtain Wikipedia-based QuitNet message vectors. We then composed term vectors for the terms that occur in QuitNet by adding QuitNet message vectors for each message in which a given term in QuitNet occurred. As such, these term vectors encode distributional information from Wikipedia and from QuitNet-specific contextual use of terms. Finally, QuitNet message vectors were generated by superposing these term vectors. This procedure is illustrated in Figure 2.

**Table 1:** Sample messages from QuitNet mapped to the thematic category in the taxonomy.

Taxonomy	Sample message snippets from QuitNet
Goals and planning	I pledge not to smoke today and extend my hand to the next quitter who drops by
Feedback and monitoring	////13 days, 23 hours, 47 minutes and 51 seconds smoke free. 280 cigarettes not smoked. $56.98 and 2 days, 3 hours of my life saved! My quit date: 2/16/2007 10:00:00 AM
Social support	Awww! Sorry you're not feeling up to par. Hope you’ll feel like joining in again soon!////Take care...////:J
Shaping knowledge	That “Demon Nic” article you copied is one/if not THE best thing I’ve ever read on the subject of quitting smoking - what an incentive!!
Natural consequences	To tell you the truth, it’s a new experience for me NOT to cough (I smoked for 38 or so years - YUK!). Good luck to you
Comparison of behavior	Thanks for the hand and I extend mine to the next in line.////XXX
Associations	I am happy to add your 7500 cancer sticks to the fire! wwwooooosssssshhhhhhhhhh Sit back and relax!
Repetition and substitution	Wow!!! xyxx is correct. You control your attitude. Deep breathing, chew gum, take a walk (or maybe a hike:-) Hang in there. This is not easy, your an addict.
Comparison of outcomes	At this point in your Quit it may be best to look at more immediate gains such as money saved or improved self esteem or better health. My dollar savings are $5,293 and that is real and for now. My life saved is an unrealized 15 weeks and 20 minutes that may never happen.
Rewards and threat	Congratulations on your Quit. In the end YOUWIN...IWIN...WEWIN!
Regulation	If anxiety is an issue for you in general or you used smoking to alleviate it, using new coping tools and behaviors to help you relax and unwind will help this symptom fade quicker. The important thing is to keep going
Antecedents	I found this site before I quit also, and it made an incredible difference in my ability to be successful. Come over to the QuitStop Forum on this site and you will meet many people with all different stories about what worked for them and what didn’t.
Identity	Great insight, YYY. But have been suffering a lot of depression type symptoms ....never occurred to me could be related to the smoking cessation. That actually helps me see things a little differently.
Scheduled consequences	Great News Also!!! After further Review, ZZZ it is now official! As of 6:00 AM you are on the Q Anni List so your 2 month quit is officially good now! The points will stay on the board! Keep up the great play calling! Hugs
Self-belief	What a change! But my not smoking wont change. My hand to you. Steve Indecision may or may not be my problem.
Covert learning	I’ll be sending 33,657 unsmoked sick sticks to a blazing end. I don’t want or need them!////I need a shot of apricot brandy and a relaxing hammock to settle in

The components of the vectors generated in this way were used as feature vectors for supervised machine learning that was conducted using the widely used Waikato Environment for Knowledge Analysis open source package for machine learning [39]. Each of the techniques of the behavior change taxonomy was used as a target for classification. Ten-fold cross-validation was applied using the naïve Bayes classifier to evaluate a binary classifier for each of the themes. Each of the trained and validated classification models was then used to classify the entire set of 2.05 million messages. Given the highly engaged users in this paper, we focused on the manifestation of behavior change techniques within the messages exchanged by 144 users identified in the earlier step, thus limiting our analysis to 159,483 messages exchanged by these users.

##### Sentiment Analysis

Sentiment analysis of user communication in QuitNet was performed using the open source software R (The R Foundation) on 159,483 messages exchanged by highly engaged users. Multiple packages were evaluated by comparing their output to manual annotation provided by 2 independent coders. Based on reliability measures, we chose the best performing package to ensure the suitability of the classification to QuitNet interactions. The SentimentAnalysis package [40] in R yielded the best results at 0.81 (average system-rater agreement measured using Cohen kappa) reaching good interrater agreement. This led to classification of messages by 144 users into the categories positive, negative, and neutral using the function *convertToDirection(sentiment$SentimentQDAP)*. The inbuilt dictionaries within the R package were used to assign a particular sentiment class to the QuitNet messages.

## Results

### Characterization of QuitNet Users

**Figure 3:**
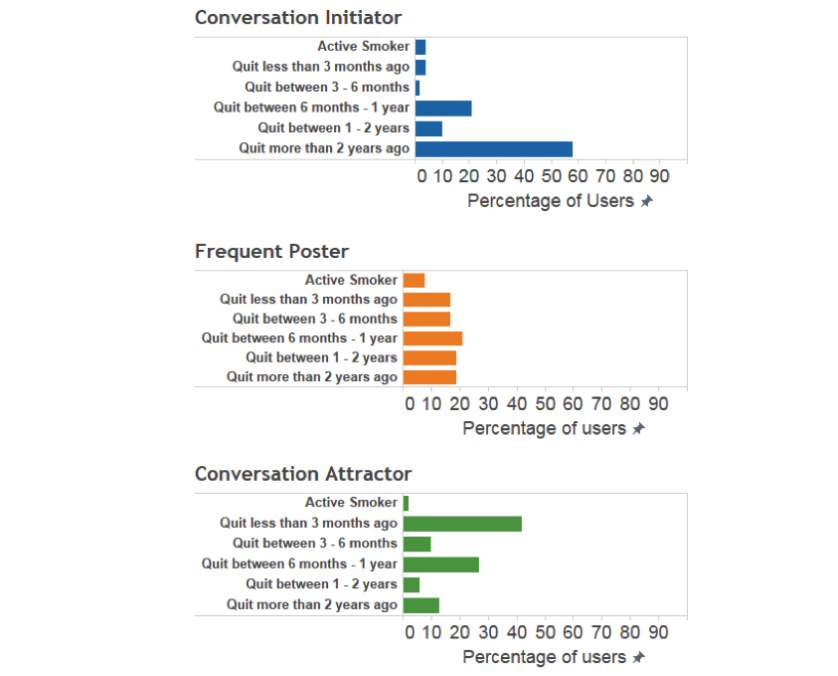
Percentages of users in each behavioral status.

The average age of the 144 QuitNet users considered in this analysis was 49 (SD 9.4) years with 82.6% (119/144) female users. Among conversation attractors, 69% (33/48) were female users with an average age of 48 (SD 10.1) years. The remaining 31% (16/48) male users had an average age of 42 (SD 6.6) years. Of the conversation initiators, 85% (41/48) were female users with an average age of 52 (SD 9.4) years. The remaining 15% (7/48) were male users with an average age of 46 (SD 6.8) years. Of the frequent posters, 94% (45/48) were female users with an average age of 49 (SD 8.6) years. The remaining 6% (3/48) were male with an average age of 51 (SD 10.2) years. Figure 3 provides the variations in smoking status across these user groups. Among conversation initiators, users who had been abstinent for more than 2 years accounted for 58% (28/48). The highest proportion of users among conversation attractors was individuals who were within 3 months of a quit attempt, with close to 42% (20/48) of the users in this category. Among frequent posters, QuitNet users with varying lengths of quit attempts had equal representation.

### Characterization of QuitNet Communication Content

Due to insufficient positive examples in the training set, we disregarded 8 of the 16 techniques of the taxonomy for final classification. For the remaining 8 techniques, the precision, recall, and f-measure for the cross-validation of the automated classification method using the naïve Bayes classifier were 0.80, 0.70, and 0.71, respectively. The themes considered for further analysis were goals and planning, feedback and monitoring, social support, natural consequences, comparison of behavior, comparison of outcomes, rewards and threat, and self-belief.

### Relationship Between Communication Content and QuitNet User Groups

#### Behavior Change Techniques

**Figure 4:**
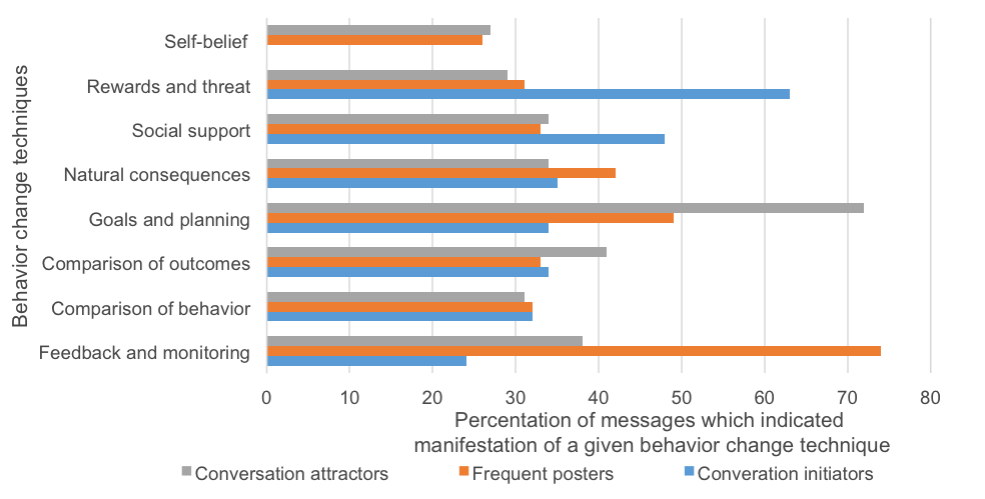
Most communicated themes among QuitNet user groups.

The average percentages of messages across different QuitNet user groups are shown in Figure 4. As seen in the figure, we observed manifestation of goals and planning and comparison of outcomes in 72.00% (19,056/26,466) and 41.00% (10,851/26,466), respectively, of the messages exchanged by conversation attractors. Among conversation initiators, the most exchanged techniques were rewards and threat and social support, in 63.00% (36,149/57,379) and 48.00% (27,542/57,379) of the messages, respectively. Among frequent posters, feedback and monitoring followed by goals and planning were embedded in 74.00% (55,972/75,638) and 49.00% (37,062/75,638) of the messages, respectively.

**Figure 5:**
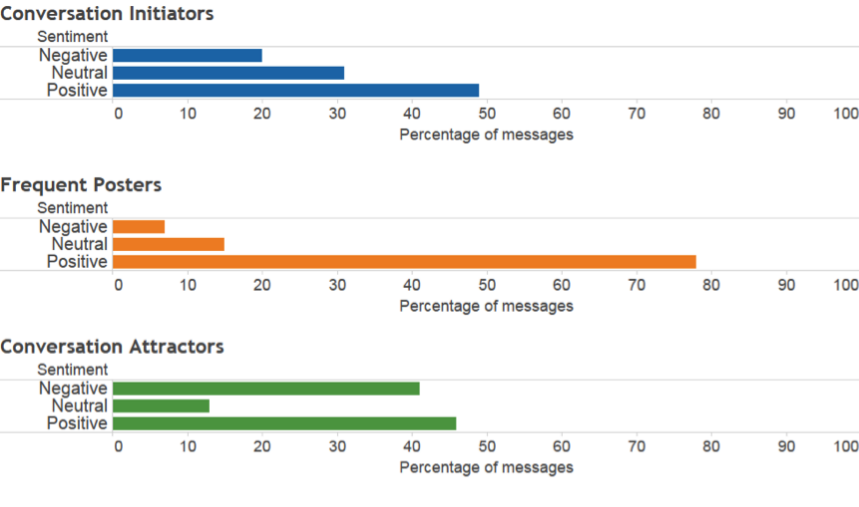
Sentiment analysis of QuitNet communication.

#### Sentiments

Considering messages exchanged by conversation attractors (see Figure 5), 45.00% (11,910/26,466) of messages were found to be containing positive sentiments, while messages with negative sentiments accounted for 40.00% (10,586/26,466). Conversation initiators’ posts were more positive, with 50.00% (28,690/57,379) of their messages classified as positive and about 23.00% (13,197/57,379) classified as negative. The frequent posters’ discussions were also positive in nature, with 78.00% (58,998/75,638) of the messages classified as containing positive sentiments.

## Discussion

### Principal Findings

In this study, we developed a novel content plus frequency framework to identify highly engaged users across 3 social categories and identify each category’s most common behavior change strategies. These methods and findings can help interventionists, technology developers, and health professionals (1) understand the techniques frequently used by highly engaged users in an online health community; (2) model, implement, and evaluate these known techniques to improve user engagement in other modalities and intervention programs; and (3) develop population health management strategies by considering the observed communication characteristics and behavioral attributes of highly engaged users.

Conversation attractors were predominantly recently quit users who appear to be facing difficulties after quitting and were seeking support from other users. They tend to discuss goals and planning and comparison of outcomes with tendencies toward negative sentiment, consistent with the communication of difficulties in the acute cessation process. Conversely, conversation initiators on QuitNet were veteran users who had been abstinent for more than 2 years. Their long duration in the community, abstinence, and high percentage of messages covering social support and rewards and threat (with a positive sentiment trend) suggests that they serve as community elders, motivating and supporting the quit attempts of newer users. Frequent posters were overwhelmingly female (96%) with no specific trend in their smoking status. The majority of their messages were positive (78%) and tended to refer to popular traditions [27] within QuitNet (such as virtual bonfire events or pledging to not smoke). In QuitNet, these techniques manifest in the form of users sharing positive and negative consequences they have encountered after quitting and pledges to not smoke for the day. Internal norms, traditions, and celebrations form a central core of engagement within QuitNet [27] and other online behavior change platforms that emphasize social support. These results suggest that identification and engagement of conversation attractors in particular may allow intervention designers to modify and enhance or even create new norms and traditions for dissemination through a community.

### Limitations

There are a number of limitations to this study. Given our primary motivation was the development of new methods, we arbitrarily set thresholds for the top engaged users. Expansion of thresholds could change the characteristics of the findings. The manual coding process of behavior change techniques was limited to 16 high-level categories, which may not fully cover the array of techniques present in QuitNet or other programs. The automated text analysis methods can be improved using advanced word representation techniques (eg, convolutional neural networks [41,42]). Finally, we used off-the-shelf sentiment analysis tools, which can be improved with n-grams, corpus-specific advanced machine learning classifiers, or specialized dictionaries [43,44].

### Conclusions

Sustained engagement with social support and effective behavior change programs remains the core principal of most online platforms that seek participation of patients in their own care. Support from peers, community-driven rituals, guidance from veteran users, and a safe and secure environment for open communication are a few motivational factors that have been hypothesized to drive engagement in QuitNet and are confirmed in this analysis. Insights from this study and future work using the same or similar framework may allow the implementation of targeted recommendation engines [45-47] to promote meaningful affiliations with content (such as information about nicotine substitutes) and connections (such as connecting users who have quit recently to veteran users) personalized based on user characteristics (eg, age, gender, smoking status). Ultimately, achieving the promise of patient participation in online communities requires the platforms themselves to evolve in response to individual and network changes and preferences that develop over time. This study offers a potential framework to drive such observation, evaluation, and ultimate change to better serve users and close the loop between intervention providers and their participants.

## Abbreviations

SGNS: Skipgram-with-Negative-Sampling

## References

[1] Frist WH (2014). Connected health and the rise of the patient-consumer. Health Aff (Millwood).

[2] Chouvarda IG, Goulis DG, Lambrinoudaki I, Maglaveras N (2015). Connected health and integrated care: toward new models for chronic disease management. Maturitas.

[3] Caulfield B, Donnelly S (2013). What is Connected Health and why will it change your practice?. QJM.

[4] Fernandez-Luque L, Vilmarlund V, Borycki E, Schulz S, Kuziemsky C, Marschollek M, Kulikowski CA (2016). Social media as catalyzer for connected health: hype or hope? Perspectives from IMIA working groups. Stud Health Technol Inform.

[5] Korda H, Itani Z (2013). Harnessing social media for health promotion and behavior change. Health Promot Pract.

[6] Eysenbach G (2008). Medicine 2.0: social networking, collaboration, participation, apomediation, and openness. J Med Internet Res.

[7] Wang X, Zuo Z, Zhao K (2015). The evolution of user roles in online health communities: a social support perspective. PACIS Proceedings.

[8] Rothert K, Strecher V, Doyle L, Caplan W, Joyce J, Jimison H, Karm LM, Mims AD, Roth MA (2006). Web-based weight management programs in an integrated health care setting: a randomized, controlled trial. Obesity (Silver Spring).

[9] Glasgow RE, Boles SM, McKay HG, Feil EG, Barrera M (2003). The D-Net diabetes self-management program: long-term implementation, outcomes, and generalization results. Prev Med.

[10] Eysenbach G (2005). The law of attrition. J Med Internet Res.

[11] Schubart JR, Stuckey HL, Ganeshamoorthy A, Sciamanna CN (2011). Chronic health conditions and internet behavioral interventions: a review of factors to enhance user engagement. Comput Inform Nurs.

[12] Michie S, Yardley L, West R, Patrick K, Greaves F (2017). Developing and evaluating digital interventions to promote behavior change in health and health care: recommendations resulting from an international workshop. J Med Internet Res.

[13] Attfield S, Kazai G, Lalmas M, Piwowarski B (2011). Towards a science of user engagement.

[14] Barello S, Graffigna G, Vegni E, Bosio A (2014). The challenges of conceptualizing patient engagement in health care: a lexicographic literature review. J Participat Med.

[15] Perski O, Blandford A, West R, Michie S (2017). Conceptualising engagement with digital behaviour change interventions: a systematic review using principles from critical interpretive synthesis. Transl Behav Med.

[16] Yardley L, Spring B, Riper H, Morrison L, Crane D, Curtis K, Merchant G, Naughton F, Blandford A (2016). Understanding and promoting effective engagement with digital behavior change interventions. Am J Prev Med.

[17] Lalmas M, O'Brien H, Yom-Tov E (2014). Measuring User Engagement (Synthesis Lectures on Information Concepts, Retrieval, and Services).

[18] Lehmann J, Lalmas M, Yom-Tov E, Dupret G (2012). Models of user engagement.

[19] Comello M, Qian X, Deal A, Ribisl K, Linnan L, Tate D (2016). Impact of game-inspired infographics on user engagement and information processing in an eHealth program. J Med Internet Res.

[20] Dijkstra A, Ballast K (2012). Personalization and perceived personal relevance in computer-tailored persuasion in smoking cessation. Br J Health Psychol.

[21] Wang X, Zhao K, Street N (2014). Social Support and user engagement in online health communities.

[22] Sun N, Rau P, Ma L (2014). Understanding lurkers in online communities: a literature review. Comput Hum Behav.

[23] Zhang S, Elhadad N (2016). Factors contributing to dropping-out in an online health community: static and longitudinal analyses.

[24] Morrison C, Doherty G (2014). Analyzing engagement in a web-based intervention platform through visualizing log-data. J Med Internet Res.

[25] Zhong C, Salehi M, Shah S, Cobzarenco M, Sastry N, Cha M (2014). Social bootstrapping: how Pinterest and Last.fm social communities benefit by borrowing links from Facebook.

[26] Zhong C, Shah S, Sundaravadivelan K, Sastry N (2013). Sharing the loves: understanding the how and why of online content curation.

[27] Myneni S, Fujimoto K, Cobb N, Cohen T (2015). Content-driven analysis of an online community for smoking cessation: integration of qualitative techniques, automated text analysis, and affiliation networks. Am J Public Health.

[28] Myneni S, Cobb N, Cohen T (2016). , & Cohen, T. Content-specific network analysis of peer-to-peer communication in an online community for smoking cessation.

[29] Sridharan V, Cohen T, Cobb N, Myneni S (2016). Characterization of temporal semantic shifts in peer-to-peer communication in a health-related online community: implications for data-driven for health promotion.

[30] Myneni S, Cobb N, Cohen T (2016). In pursuit of theoretical ground in behavior change support systems: analysis of peer-to-peer communication in a health-related online community. J Med Internet Res.

[31] Mittler J, Martsolf G, Telenko S, Scanlon D (2013). Making sense of “consumer engagement” initiatives to improve health and health care: a conceptual framework to guide policy and practice. Milbank Q.

[32] Short C, Rebar A, Plotnikoff R, Vandelanotte C (2015). Designing engaging online behavior change interventions: a proposed model of user engagement. Eur Psychol.

[33] QuitNet LLC.

[34] Cobb N, Graham A, Bock B, Papandonatos G, Abrams D (2005). Initial evaluation of a real-world Internet smoking cessation system. Nicotine Tob Res.

[35] Michie S, Richardson M, Johnston M, Abraham C, Francis J, Hardeman W, Eccles MP, Cane J, Wood CE (2013). The behavior change technique taxonomy (v1) of 93 hierarchically clustered techniques: building an international consensus for the reporting of behavior change interventions. Ann Behav Med.

[36] Mikolov T, Sutskever L, Chen K, Corrado G, Dean J (2013). Distributed representations of words and phrases and their compositionality.

[37] Widdows D, Cohen T (2010). The semantic vectors package: new algorithms and public tools for distributional semantics.

[38] Cohen T, Schvaneveldt R, Widdows D (2010). Reflective Random Indexing and indirect inference: a scalable method for discovery of implicit connections. J Biomed Inform.

[39] Hall M, Frank E, Holmes G, Pfahringer B, Reutemann P, Witten I (2009). The WEKA data mining software: an update. IGKDD Explorations.

[40] Sentiment analysis package.

[41] Zhang S, Grave E, Sklar E, Elhadad N (2017). Longitudinal analysis of discussion topics in an online breast cancer community using convolutional neural networks. J Biomed Inform.

[42] Zhang ML, Zhou ZH (2006). Multilabel neural networks with applications to functional genomics and text categorization. IEEE Transactions on Knowledge and Data Engineering.

[43] Da Silva NF, Hruschka ER, Hruschka Jr ER (2014). Tweet sentiment analysis with classifier ensembles. Decision Support Systems.

[44] Pröllochs N, Feuerriegel S, Neumann D (2015). Generating domain-specific dictionaries using Bayesian learning. InECIS.

[45] Pazzani M, Billsus D (2007). Content-based recommendation systems. The Adaptive Web.

[46] Melville P, Raymond J, Nagarajan R (2002). Content-boosted collaborative filtering for improved recommendations.

[47] Hannon J, Bennett M, Smyth B (2010). Recommending twitter users to follow using contentcollaborative filtering approaches.

